# Comparison of clinicopathological characteristics and survival between symptomatic and asymptomatic anaplastic thyroid carcinoma

**DOI:** 10.1038/s41598-023-30162-5

**Published:** 2023-02-24

**Authors:** Seomin Cho, Haejung Kim, Young Lyun Oh, Soo Yeon Hahn, Tae Hyuk Kim, Jung Hee Shin

**Affiliations:** 1 Sungkyunkwan University School of Medicine, Suwon, Republic of Korea; 2 Department of Radiology and Center for Imaging Science, Samsung Medical Center, Sungkyunkwan University School of Medicine, 81, Irwon-ro, Gangnam-gu, Seoul, 06351 Republic of Korea; 3 Department of Pathology, Samsung Medical Center, Sungkyunkwan University School of Medicine, Seoul, Republic of Korea; 4 Division of Endocrinology and Metabolism, Department of Medicine, Samsung Medical Center, Sungkyunkwan University School of Medicine, Seoul, Republic of Korea

**Keywords:** Diseases, Oncology

## Abstract

Although anaplastic thyroid carcinoma (ATC) is a fatal form of thyroid cancer with an overall survival of only a few months, there are some factors associated with longer survival. However, it remains unknown whether asymptomatic ATC differs from symptomatic ATC in terms of characteristics and overall prognosis. Therefore, we aimed to examine the clinicopathological characteristics and prognosis of asymptomatic ATC compared with those of symptomatic ATC. We retrospectively reviewed the medical records of 113 patients with ATC who were registered at our institution between November 1994 and July 2020. A total of 86 patients (59 women and 27 men; mean age, 66.9 ± 11.1 years) were enrolled for analysis. The clinicopathological characteristics of the ATC cohort were evaluated, and prognostic factors associated with disease-specific mortality were assessed. Of the 86 patients with ATC, 78 were symptomatic and eight were asymptomatic. Compared with the symptomatic group, the asymptomatic group had a younger age at diagnosis (59.3 ± 10.3 vs. 67.7 ± 11.0 years, *p* = 0.045), smaller tumor size (2.8 ± 1.2 vs. 5.8 ± 2.0 cm, *p* < 0.001), and longer survival period (37.5 ± 46.4, 9.5 ± 16.8 months, *p* < 0.001). However, the ATC component (%) of the tumor, sex, ultrasonographic risk category, and distant metastasis at diagnosis did not differ significantly between the two groups. In the multivariate Cox regression analysis, asymptomatic ATC (HR: 0.33, 95% CI 0.11–0.99, *p* = 0.045) and absence of distant metastasis (hazard ratio (HR): 0.56, 95% Confidence interval (CI) 0.35–0.88, *p* = 0.012) were associated with longer survival. Patients with asymptomatic ATC have a smaller tumor size, a longer survival period, and a younger age than those with symptomatic ATC. Being asymptomatic and having no distant metastasis were associated with longer survival in patients with ATC in a clinical setting.

## Introduction

Anaplastic thyroid carcinoma (ATC) is a rare but aggressive malignancy. It accounts for only 2% of total thyroid carcinomas but contributes up to 50% of mortality^1^. The median life expectancy of patients with ATC is 4 months, and its disease-specific mortality rate reaches 100%^2^.

Despite these dire figures, there are some favorable prognostic factors associated with ATC. Specifically, prior studies have revealed that younger age, smaller tumor size, non-invasion or microinvasion, and smaller components of ATC are associated with longer survival^3–6^. However, whether asymptomatic ATC differs from symptomatic ATC in terms of clinical characteristics or overall prognosis has not been sufficiently investigated.

Asymptomatic ATC is a rare form of ATC because its distinctive characteristic is that it is a rapidly exacerbating symptom. Unlike well-differentiated thyroid carcinomas, which usually present with asymptomatic thyroid nodules, most ATC patients present with a history of fast-growing neck mass, dyspnea, dysphagia, or voice change within a few months^1^. Hence, asymptomatic ATC could potentially be considered a different disease entity in terms of prognosis.

Similar studies have been performed on papillary thyroid carcinoma (PTC), where treatment outcomes and survival rates were compared between asymptomatic and symptomatic PTCs. These studies found that asymptomatic PTC is associated with lower recurrence-free survival and higher overall survival rates than symptomatic PTC^7,8^. This finding highlights the importance of elucidating whether individuals with asymptomatic ATC might expect better disease course. Therefore, this study aimed to retrospectively examine the clinicopathological characteristics of a cohort of patients with ATC, compare them with those of patients with asymptomatic ATCs, and assess features associated with long-term survival in patients with ATC.

## Results

### Clinicopathological characteristics of ATC

In this study, 86 patients (59 women and 27 men; mean age, 66.9 ± 11.1 years; median age, 68 [36–39] years) were included. Among them, 41 (48%) patients were diagnosed surgically, 20 (23%) patients were diagnosed by US-guided FNA, and 25 (29%) patients were diagnosed by US-guided CNB. The mean survival period was 12.1 ± 22.4 months, and the median survival period was 5 (1–150) months. The 1-year survival rate was 26% (22/86 patients). The mean tumor size was 5.5 ± 2.1 cm, with a median diameter of 5.2 cm (0.9–13 cm).

The ATC component was available in 24 patients (28%) and was unavailable in the remaining cases. The mean ATC component was 76 ± 32.8%, with a median of 95% (range 10–100%). Forty-one patients (48%) had distant metastases at the time of diagnosis. K-TIRADS data were not available for three patients because US images were not obtained in those cases. In the 83 patients whose US images were available, all tumors were diagnosed as K-TIRADS 4 (intermediate suspicion) or 5 (high suspicion). Fifty-four of these 83 patients (65%) had K-TIRADS 5. The K-TIRADS did not show large variations.

The test for BRAF mutation was performed in 22 (25.6%) of 86 patients. BRAF mutation was identified in 12 (54.5%) of 22 patients.

### Comparison of clinicopathological characteristics between symptomatic and asymptomatic ATC patients

**Figure 1:**
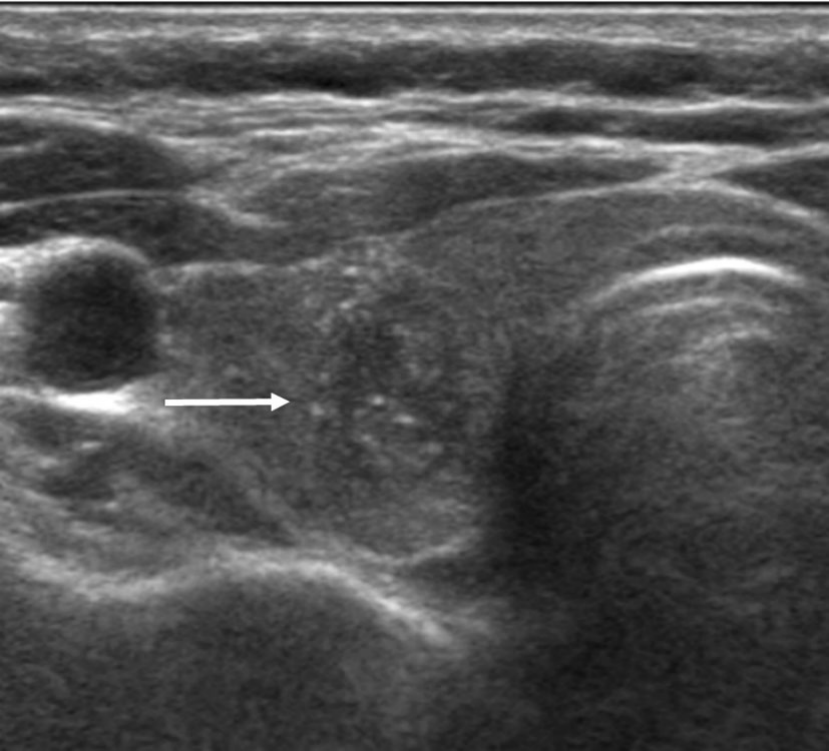
Asymptomatic anaplastic thyroid carcinoma in a 52-year-old woman. A transverse sonogram shows a 1.5-cm nonparallel oriented hypoechoic nodule (arrow) with echogenic dots in the right thyroid gland, indicating K-TIRADS 5.

**Figure 2:**
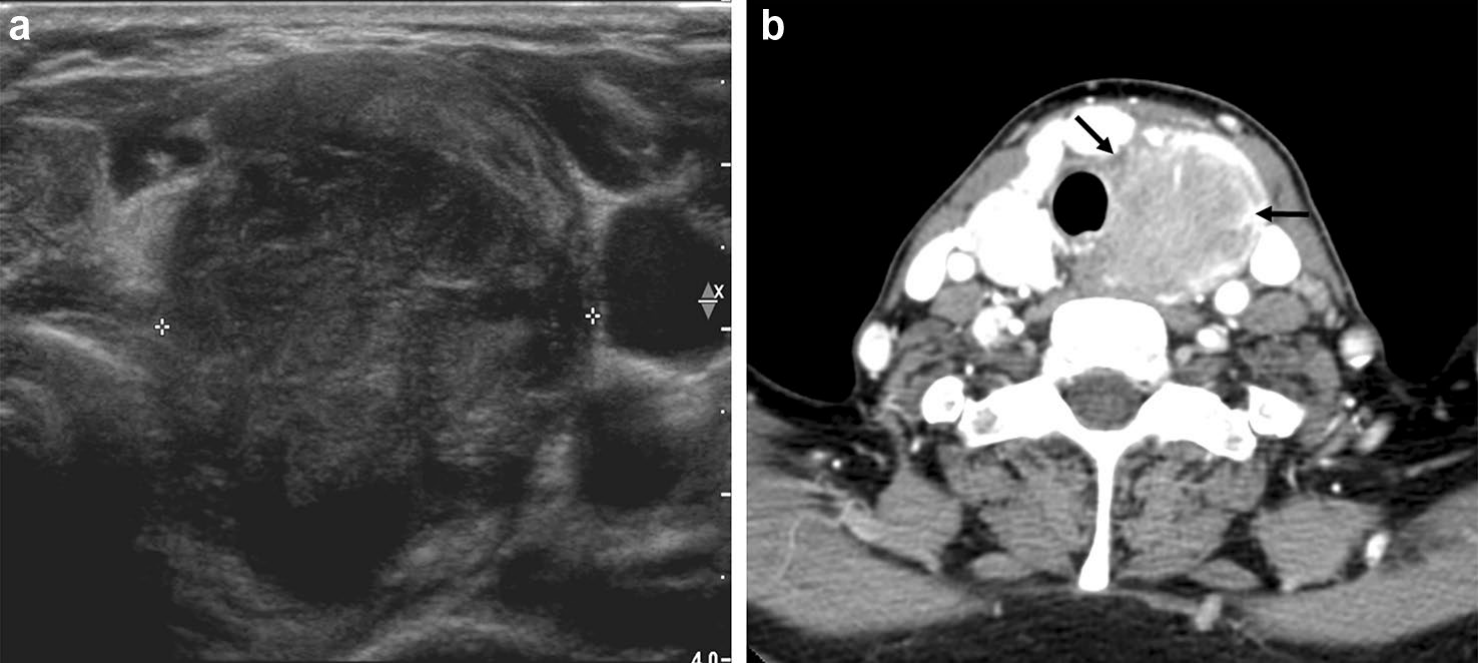
Symptomatic anaplastic thyroid carcinoma in a 58-year-old woman with swallowing difficulty. A transverse (**a**) sonogram shows a 3.4-cm irregular hypoechoic nodule (crosses) in the left thyroid gland, which was considered K-TIRADS 5. CT (**b**) reveals a heterogeneous enhanced mass (arrows) in the left thyroid gland involved in the tracheoesophageal groove.

Of the 86 patients, 78 were symptomatic and eight were asymptomatic at diagnosis. Variables that showed statistically significant differences between the two groups included age, survival period, and tumor size (Table 1). The mean age of asymptomatic ATC patients was lower than that of symptomatic ATC patients (59.3 years vs. 67.7 years, *p* = 0.045). Survival was better in asymptomatic ATC patients, with a mean survival period of 37.5 months compared to 9.5 months for symptomatic ATC patients (*p* < 0.001). The 1-year survival rate was also comparable between the two groups, with asymptomatic ATC patients having a higher 1-year survival rate (100% vs. 18%, *p* < 0.001). The mean tumor size was smaller in asymptomatic ATC patients (2.8 cm vs. 5.8 cm, *p* < 0.001). However, sex, distant metastasis, ATC component, and K-TIRADS category did not significantly differ between the two groups (Figs. 1, 2).

**Table 1:** Clinicopathological characteristics of symptomatic and asymptomatic ATC patients.

	Total (N = 86)	Symptomatic ATC (N = 78)	Asymptomatic ATC (N = 8)	p value^a^
Sex [n (%)]				
Female	59 (69)	53 (68)	6 (75)	1
Male	27 (31)	25 (32)	2 (25)	
Age (years)				
Mean ± STDV	66.9 ± 11.1	67.7 ± 11.0	59.3 ± 10.3	0.045
Median (range)	68 (36–90)	70 (36–90)	59.5 (46–72)	
Survival period (months)				
$\ge$ 1 year [*n* (%)]	22 (26)	14 (18)	8 (100)	< 0.001
Mean ± STDV	12.1 ± 22.4	9.5 ± 16.8	37.5 ± 46.4	< 0.001
Median (range)	5 (1–150)	4.5 (1–104)	20 (12–150)	
Tumor size (cm)				
Mean ± STDV	5.5 ± 2.1	5.8 ± 2.0	2.8 ± 1.2	< 0.001
Median (range)	5.2 (0.9–13)	5.5 (1.5–13)	2.7 (0.9–4.5)	
Distant metastasis [n (%)]				
Absent	45 (52)	39 (50)	6 (75)	0.270
Present	41 (48)	39 (50)	2 (25)	
ATC component (%)	(N = 24)^b^	(N = 17)	(N = 7)	
Mean ± STDV	76 ± 32.8	81 ± 30.9	64 ± 36.9	0.415
Median (range)	95 (10–100)	100 (10–100)	70 (20–100)	
K-TIRADS [*n* (%)]	(N = 83)^c^	(N = 75)	(N = 8)	
4	29 (35)	25 (33)	4 (50)	0.441
5	54 (65)	50 (67)	4 (50)	

^a^Comparison between symptomatic and asymptomatic ATC using Wilcoxon Rank-Sum Test for continuous variables (age, overall survival, tumor size and ATC component) and Fisher’s exact test for categorical variables (sex, 1-year survival, distant metastasis, K-TIRADS).

^b,c^The number of study patients differed in ATC component and K-TIRADS due to missing data.

Treatment type in the ATC patients is mentioned in the Table 2. Tracheostomy was performed in 9 patients and airway stent insertion was performed in 2 patients.

**Table 2:** Multimodal treatment type in the study patients with ATC.

Treatment type	Total (N = 86)	Symptomatic ATC (N = 78)	Asymptomatic ATC (N = 8)
OP	15	13 (16.7%)	2 (25.0%)
OP + RAI	2	0	2 (25.0%)
OP + RT	10	10 (12.8%)	0
OP + RT + Chemo (or TKI)	11	8 (10.3%)	3 (37.5%)
OP + Chemo	3	2 (2.6%)	1 (12.5%)
OP + RAI + RT + Chemo (or TKI)	2	2 (2.6%)	0
RT	5	5 (6.4%)	0
RT + Chemo (or TKI)	11	11(14.1%)	0
Chemo	1	1(1.3%)	0
No treatment	26	26 (33.3%)	0

*OP* operation, *RAI* radioiodine therapy, *RT* Radiotherapy, *Chemo* Chemotherapy, *TKI* tyrosine kinase inhibitor therapy.

### Evaluation of clinicopathological characteristics associated with survival period

On univariate Cox regression analysis, age (*p* = 0.045), state of symptoms (asymptomatic or symptomatic, *p* < 0.001), tumor size (*p* < 0.001), and distant metastasis (absent or present, *p* < 0.001) were associated with survival. On multivariate Cox analysis, the state of symptoms and distant metastasis were independent predictors of survival. Asymptomatic ATC (hazard ratio (HR): 0.33, 95% confidence interval (CI), *p* = 0.045) and the absence of distant metastasis (HR: 0.56, 95% CI = 0.35–0.88, *p* = 0.012) were associated with longer survival.

**Figure 3:**
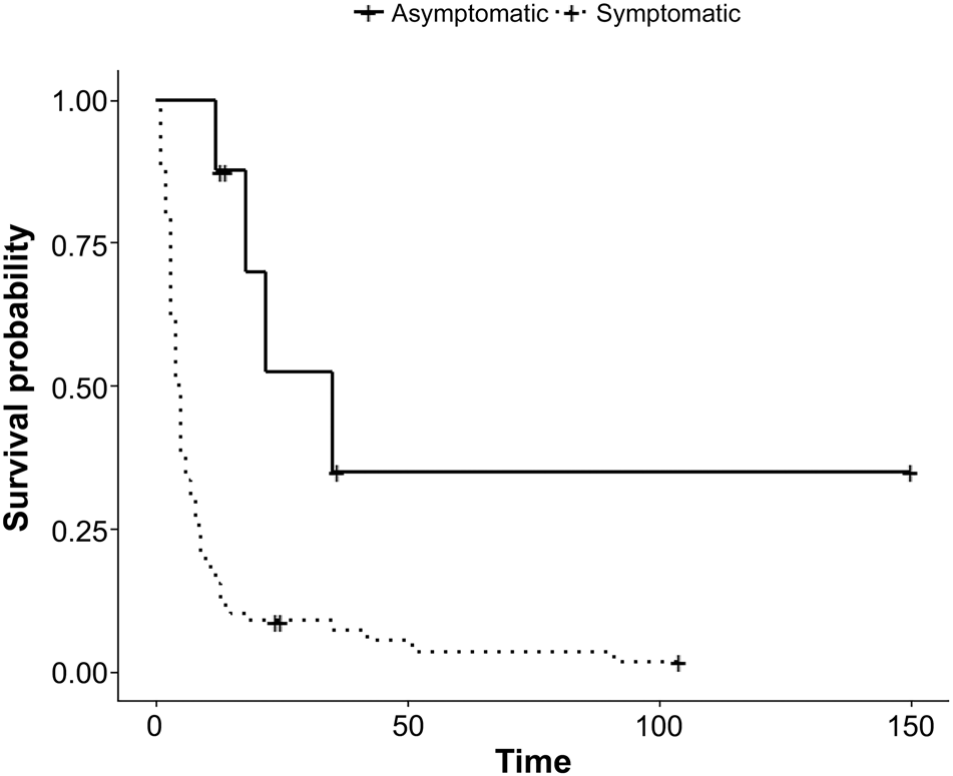
Kaplan–Meier curves for survival probability by state of symptom (asymptomatic or symptomatic) in ATC. Log-rank test, *p* < 0.001.

**Figure 4:**
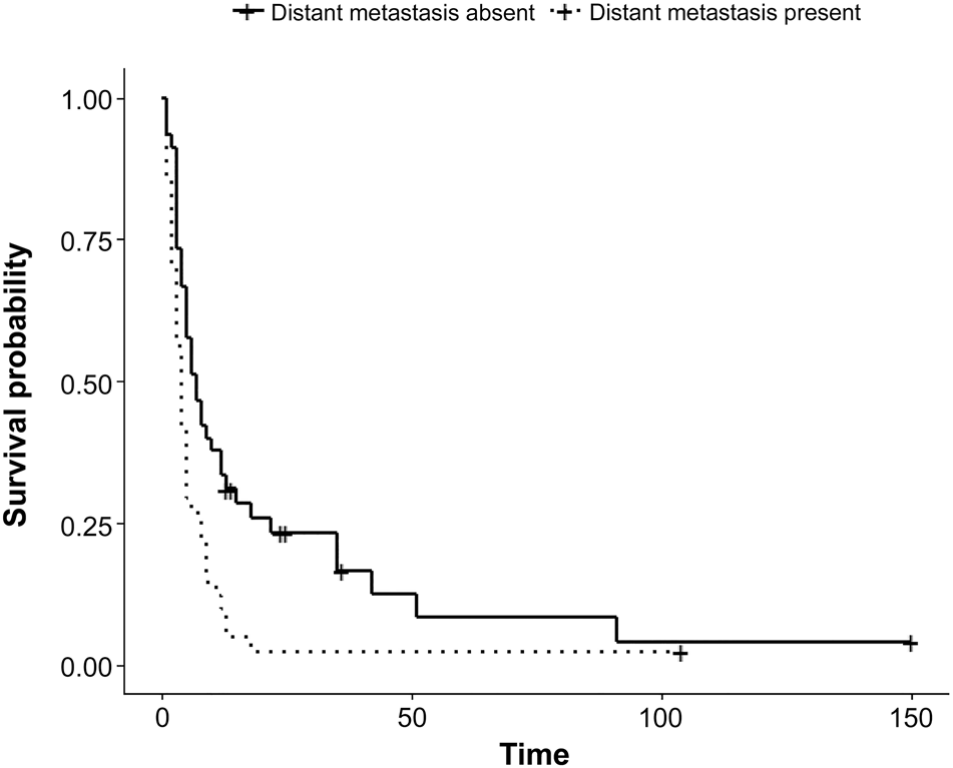
Kaplan–Meier curves for survival probability by distant metastasis (absent or present) at diagnosis in ATC. Log-rank test, *p* = 0.003.

Based on the results of the multivariate Cox analysis, the Kaplan–Meier curve is depicted in Figs. 3 and 4. A log-rank test was performed, with *p* < 0.001 for the state of symptoms and *p* = 0.003 for distant metastasis (Table 3).

**Table 3:** Multivariate cox proportional hazards regression model.

Variables	Univariate			Multivariate		
	HR	95% CI	*p* value^a^	HR	95% CI	*p* value
Sex			0.117			
Female	0.69	0.43–1.10				
Male	1.46	0.91–2.35				
Age	1.03	1.00–1.05	0.035	1.02	1.00–1.05	0.085
State of symptom			0.002			0.045
Asymptomatic	0.20	0.07–0.55		0.33	0.11–0.99	
Symptomatic	5.06	1.83–13.97		3.02	1.01–9.03	
Size	1.17	1.05–1.29	0.003	1.13	0.99–1.28	0.068
Distant metastasis			0.003			0.012
Absent	0.50	0.32–0.79		0.56	0.35–0.88	
Present	2.00	1.27–3.14		1.79	1.13–2.82	
ATC component	1.01	1.00–1.03	0.073			
K-TIRADS			0.549			
4	1.15	0.72–1.84				
5	0.87	0.54–1.38				

## Discussion

It is well known that early detection of malignant tumors in various organs leads to better prognosis^9–11^. To the best of our knowledge, there are no studies in the literature discussing whether early detection in asymptomatic status is associated with survival in patients with aggressive tumors such as ATC. Our results revealed that survival rates in asymptomatic ATC patients without metastasis were better than those with symptomatic ATC.

Owing to the popularization of screening programs and easier access to various imaging modalities and diagnostic techniques, the chance of detecting small and early stages of ATC has increased^3^. This can also be interpreted as increasing detection of ATC as an incidentaloma, considering that asymptomatic ATC has a smaller tumor size. Consequently, with the increasing chance of detecting asymptomatic ATC, which has a relatively longer survival, it seems necessary to have a different approach in how asymptomatic ATC and classical ATC are treated.

Our results also showed that asymptomatic ATC patients were younger, had smaller tumors, and had longer survival periods than symptomatic ATC patients. ATC can arise de novo or from the malignant transformation of pre-existing, well-differentiated tumors^12^. It appears likely that asymptomatic ATC is more likely to arise de novo when considering its smaller size and its patients’ generally younger age. According to our results, asymptomatic ATC requires active surgical treatment, and these patients do not need to be as concerned with their condition as do patients with symptomatic ATC. However, a study comparing the prognosis of asymptomatic ATC with high-grade, well-differentiated tumors is needed.

In multivariate Cox regression analysis, asymptomatic status and absence of distant metastasis were important long-term survival factors. This finding concurs with the new 8th edition of the AJCC classification, where the presence of distant metastasis in ATC was classified as the worst from stage IV and stage IVC, irrespective of the tumor and nodal status^13^. Taken together, asymptomatic ATC shares clinicopathological characteristics with previously described favorable prognostic factors for ATC. This suggests that asymptomatic ATC may be a phenotype of the sum of favorable prognostic factors.

We did not compare the treatment types of ATC patients because of heterogeneous trial and a long study period. A report showed that multimodal treatment including tyrosine kinase inhibitor therapy demonstrated prolonged survival with dabrafenib plus trametinib as the most effective therapeutic option demonstrated for BRAF mutant ATC patients^14^.

As ATC is a rare disease, there is an inevitable limitation in assessing prognosis. A limitation of our study is that it was retrospective. We relied on pathological reports. Furthermore, there were too many missing data points for the ATC component for us to conduct a full analysis, as surgically confirmed cases comprised only 48% of cases studied here. This made it difficult to determine the statistical significance of our findings. We expect to investigate this in a future prospective study.

## Conclusion

Patients with asymptomatic ATC had a smaller tumor size, had a longer survival period, and tended to be younger than those with symptomatic ATC. Being asymptomatic and having no distant metastasis were associated with longer survival for patients with ATC. At present, there are no specific treatment guidelines for asymptomatic ATC despite its better disease course. We suggest considering asymptomatic ATC as a separate disease entity from classical ATC.

## Methods

### Study population and data acquisition

This retrospective study was approved by the institutional review board of Samsung Medical Center and the requirement for informed consent was waived. A total of 113 ATC patients registered in our hospital from November 1994 to April 2020 were identified. Among them, 18 cases registered as post-operation status and 4 cases of recurrent disease were excluded. Additionally, 4 cases without pathology report, and one metastatic lymph node case without a known primary mass were also excluded. Finally, total 86 patients (59 females and 27 males with the mean age of 66.9 ± 11.1 years) were enrolled for the analysis. Data were obtained from electronic medical records and reviewed retrospectively.

### Definition of asymptomatic and symptomatic ATC

Asymptomatic ATC was defined as a tumor that had no signs of disease or symptoms at presentation but was incidentally detected during clinical investigation of an unrelated condition, via ultrasonography or other modalities, and histopathologically confirmed ATC. Symptomatic ATC was defined as a tumor detected based on symptoms reported by patients or physicians, such as a palpable neck mass, dyspnea, dysphagia, or voice change, and confirmed by subsequent imaging modalities and pathologic results.

### Variables

The clinicopathological features considered in the study included age at diagnosis, sex, state of symptoms (symptomatic or asymptomatic), survival period, tumor size, ATC component (%) of the tumor, presence of distant metastasis at diagnosis, and ultrasonographic (US) risk category. Survival period was calculated as the number of months from the date of diagnosis to the date of death or, in the case of surviving patients, to July 2021, which is when data collection was conducted. Tumor size was determined as the longest diameter measured by pathology. The ATC component was defined as the proportion of the total tumor mass consisting of ATC. Distant metastasis was considered positive if it was detected on examination. If distant metastasis was not detected on examination or was not verified, it was considered negative.

The Thyroid Imaging Reporting and Data System (K-TIRADS) was used to determine the US risk category. K-TIRADS classifies a thyroid nodule into one of five categories according to its US patterns and malignancy risk. The categories are as follows: 1, no nodule, 2; benign, 3; low suspicion; 4, intermediate suspicion; 5, high suspicion^15^. The molecular test for BRAF mutation was assessed. Treatment type of ATC patients was evaluated.

### Ultrasonographic evaluation

All US examinations were performed by one of seven radiologists. US machines with a linear probe (HDI 5000 or iU22; Phillips Healthcare) were used. A prospective review of the US images in all patients was performed by an experienced thyroid radiologist (S.J.H). The lesions were assessed based on K-TIRADS categories.

### Pathologic analysis

Generally, ATC has three main histological growth patterns: spindle cell pattern, pleomorphic giant cell pattern, and squamoid pattern. One of these patterns may predominate in a given tumor or the tumor may comprise two or three different patterns^16^.

Pathologic reports written by one of seven pathologists were evaluated for tumor size and the ATC component of the tumor. All thyroid tumors with any ATC component were diagnosed as ATC, regardless of the proportion of ATC in the tumor. In some cases, immunohistochemical staining was performed to confirm the diagnosis. However, the percentage of ATC components was described from in 2012 at our institution, and the percentage was not proven in cases where diagnoses were made solely by US-guided fine-needle aspiration (FNA) or core needle biopsy (CNB) instead of thyroidectomy.

### Statistical analysis

Continuous variables are expressed as mean ± standard deviation or median (range). Categorical variables are expressed as numbers and frequencies as percentages. Given the small sample size due to the rarity of this disease, we opted to use the Wilcoxon rank-sum test and Fisher’s exact test to compare differences between the symptomatic and asymptomatic groups. A univariate Cox regression model was used to detect variables associated with survival period. Prior to Cox regression analysis, scaled Schoenfeld residuals were used to test the proportional hazards assumption. Variables that showed a statistically significant association in the univariate Cox analysis were included in multivariate Cox analysis. Overall survival of variables that were statistically significant in multivariate Cox regression analysis was depicted using the Kaplan–Meier method. The log-rank test was used to determine statistical significance. For all analyses, two-sided *p* values < 0.05 were considered statistically significant. These analyses were performed using the R programming studio (R Foundation for Statistical Computing, Vienna, Austria).

## Data Availability

The datasets used and/or analysed during the current study available from the corresponding author on reasonable request. All methods were performed in accordance with the relevant guidelines and regulations by including a statement in the methods section to this effect.
